# Cardiac Stroke Volume Index Is Associated With Early Neurological Improvement in Acute Ischemic Stroke Patients

**DOI:** 10.3389/fphys.2021.689278

**Published:** 2021-11-18

**Authors:** Joseph Miller, Farhan Chaudhry, Sam Tirgari, Sean Calo, Ariel P. Walker, Richard Thompson, Bashar Nahab, Christopher Lewandowski, Phillip Levy

**Affiliations:** ^1^Department of Emergency Medicine and Internal Medicine, Henry Ford Hospital and Wayne State University, Detroit, MI, United States; ^2^Department of Emergency Medicine and Integrative Biosciences Center, Wayne State University, Detroit, MI, United States; ^3^Central Michigan University College of Medicine, Mount Pleasant, MI, United States; ^4^Department of Anesthesiology, University of California, San Francisco, San Francisco, CA, United States; ^5^Department of Radiology, Harvard Medical School, Cambridge, MA, United States; ^6^Department of Emergency Medicine, Henry Ford Hospital and Wayne State University, Detroit, MI, United States

**Keywords:** ischemic stroke (IS), cardiac function, heart brain interaction, autonomic dysfunction, stroke outcome and recovery

## Abstract

Early neurological improvement as assessed with the NIH stroke scale (NIHSS) at 24 h has been associated with improved long-term functional outcomes following acute ischemic stroke (AIS). Cardiac dysfunction is often present in AIS, but its association with outcomes is incompletely defined. We performed a pilot study to evaluate the association between non-invasively measured cardiac parameters and 24-h neurological improvement in prospectively enrolled patients with suspected AIS who presented within 12 h of symptom-onset and had an initial systolic blood pressure>140 mm Hg. Patients receiving thrombolytic therapy or mechanical thrombectomy were excluded. Non-invasive pulse contour analysis was used to measure mean arterial blood pressure (MAP), cardiac stroke volume index (cSVI), cardiac output (CO) and cardiac index (CI). Transcranial Doppler recorded mean middle cerebral artery flow velocity (MFV). We defined a decrease of 4 NIHSS points or NIHSS ≤ 1 at 24-h as neurological improvement. Of 75 suspected, 38 had confirmed AIS and did not receive reperfusion therapy. Of these, 7/38 (18.4%) had neurological improvement over 24 h. MAP was greater in those without improvement (108, IQR 96–123 mm Hg) vs. those with (89, IQR 73–104 mm Hg). cSVI, CO, and MFV were similar between those without and with improvement: 37.4 (IQR 30.9–47.7) vs. 44.7 (IQR 42.3–55.3) ml/m^2^; 5.2 (IQR 4.2–6.6) vs. 5.3 (IQR 4.7–6.7) mL/min; and 39.9 (IQR 32.1–45.7) vs. 34.4 (IQR 27.1–49.2) cm/s, respectively. Multivariate analysis found MAP and cSVI as predictors for improvement (OR 0.93, 95%CI 0.85–0.98 and 1.14, 95%CI 1.03–1.31). In this pilot study, cSVI and MAP were associated with 24-h neurological improvement in AIS.

## Introduction

Acute ischemic stroke (AIS) is the leading cause of long-term disability and results in worsening functional independence long after initial stroke (Dhamoon et al., 2009; Virani et al., 2020). Twenty-hour neurological improvement, as assessed by the National Institute of Health Stroke Scale (NIHSS), is associated with good long-term functional outcomes following acute stroke (Takagi et al., 2014; Rangaraju et al., 2016; Wouters et al., 2018). Rapid reperfusion strategies have been implemented to rapidly restore blood flow to the penumbra resulting in improved outcomes; however, many patients do not have access to these strategies and/or do not qualify for reperfusion (Bhaskar et al., 2018). Therefore, identification of modifiable factors associated with 24-h neurological improvement in non-reperfused patients could inform their management and prognosis.

AIS diminishes the cerebrovascular autoregulation, thus penumbra blood flow becomes directly dependent on cardiac function (Tranmer et al., 1992). Likewise, AIS can result in sympathetic activation and impaired parasympathetic tone resulting in stroke-induced heart injury, characterized by LV dysfunction (Wrigley et al., 2017; Sposato et al., 2020). Therefore, identifying pertinent aspects of cardiac function associated with 24-h neurological improvement following AIS may have prognostic and acute management implications. Given the acuity associated with AIS management, measurements of cardiac function need to be quick and non-invasive, especially if they are performed on arrival in the emergency department (ED). For this brief research report, we conducted a pilot prospective observational study of AIS patients using a non-invasive monitoring device to test the feasibility of rapidly assessing various cardiac parameters in association with 24-h neurological outcomes.

## Materials and Methods

### Enrollment

We conducted this prospective, observational study of AIS patients at a large, urban, ED, which is part of a comprehensive stroke hospital, Henry Ford Hospital, from July 2014 through September 2016. The study was approved by the hospital’s IRB and registered at ClinicalTrials.gov (NCT02056821). We enrolled patients 18–90 years old with suspected AIS presenting within 12-h of symptom onset and with a systolic blood pressure>140 mmHg. AIS was confirmed if symptoms lasted more than 24-h or less than 24 h with ischemic lesion on diffusion-weighted imaging. Exclusion criteria included baseline modified Rankin Scale>3, pregnancy, intracranial hemorrhage on computed tomography, treatment with thrombolytic or mechanical thrombectomy, advanced directive for comfort care/hospice, or requiring endotracheal intubation.

### Data Collection

Trained investigators obtained consent and documented baseline demographic and clinical characteristics including age, sex, and past medical history. Localization of stroke was divided into lacunar vs. non-lacunar stroke. Patient treatment with intravenous (IV) fluids and IV antihypertensive were also recorded. NIHSS was documented upon arrival by the stroke neurology team and was then confirmed by an investigator prior to enrollment.

### Hemodynamic

Hemodynamic variables were measured with the clinically validated (Nexfin device, Edwards Lifescience, Irvine, CA) (Broch et al., 2012; Martina et al., 2012). This novel non-invasive monitor uses pulse-contour analysis to determine multiple hemodynamic parameters including mean arterial blood pressure (MAP), cardiac stroke volume index (cSVI), cardiac output (CO), and cardiac index (CI). Upon enrollment, a member of the research team placed the device on a non-paretic upper extremity to record beat-to-beat hemodynamic data for 4 h. A trained technician also performed transcranial Doppler imaging (TCD) on all patients looking for middle cerebral artery (MCA) mean flow velocity (MFV) on the affected-side. We averaged all TCD and hemodynamic values over 5-min periods.

### Primary Outcome

NIHSS was calculated on arrival and after 24-h. Neurological improvement was defined as a decrease of 4 or more points on the NIHSS or a score equal to or less than one at 24-h (improvement) (National Institute of Neurological Disorders and Stroke rt-Pa Stroke Study Group, 1995).

### Statistical Analysis

We reported continuous variables as median with interquartile range (IQR) and binomial variables as counts with percentages (%). Wilcoxon-Mann Whitney test and Fisher’s-exact test was performed to compare continuous and categorical variables, respectively, between improvement vs. no improvement. We used a univariate logistic regression model on all identified variables assessing association with 24-h neurologic improvement. We then performed a multivariate logistic regression model to determine which variables were independently associated with 24-h neurologic improvement. Variables were selected using a stepwise logistic regression minimizing the Akaike-information-criterion. We used McFadden pseudo-R^2^ to assess the model by quantifying the proportion of the total variability on the outcome from the variables (Louapre et al., 2020). Regression models used 100-iterations maximum to reach convergence. Results were reported as odds ratios (ORs) with 95% confidence intervals (CI). A 2-sided *P*-value < 0.05 was considered statistically significant. Analysis was completed with R-version 3.6.3.

## Results

### Patient Enrollment

Seventy five patients met enrollment criteria with suspected AIS, but only 55 were confirmed stroke. We were unable to obtain hemodynamic measurements on 7 patients due to inadequate recording by the device, and 10 patients received thrombolytic therapy and were also removed, resulting in 38 patients for further analysis.

### Baseline Characteristics

7/38 (18.4%) patients showed signs of 24-h neurologic improvement (Table 1). There was no significant difference in age, sex, African American race, body mass index (BMI), history of hypertension, coronary artery or peripheral vascular disease (CAD/PVD), diabetes mellitus, transient ischemic attack (TIA)/stroke, anticoagulation use or smoking were noted between those with or without improvement.

**TABLE 1:** Patient demographic, clinical, and hemodynamic data with univariate and multivariate results.

(A) Univariate	All (*n* = 38)	No Improvement (*n* = 31)	Improvement (*n* = 7)	OR	95%CI	*P*-value
**Patient characteristics**						
Age in years (IQR)	66.5 (57–73)	67 (58–73.5)	63 (52–70.5)	0.979	0.916–1.05	0.524
Male sex (%)	17/38 (44.7)	14/31 (45.2)	3/7 (42.9)	0.911	0.157–4.81	0.911
African American (%)	28/38 (73.7)	22/31 (71.0)	6/7 (85.7)	0.407	0.0200–2.89	0.435
BMI, kg/m^2^ (IQR)	28.1 (23.9–31.0)	28.5 (23.4–33.4)	27.4 (26.6–29.3)	0.976	0.844–1.12	0.735
**Past medical history**						
Hypertension (%)	32/38 (84.2)	26/31 (83.9)	6/7 (85.7)	1.15	0.146–24.2	0.904
CAD/PVD (%)	7/38 (18.4)	6/31 (19.4)	1/7 (14.3)	0.694	0.0333–5.26	0.756
Diabetes Mellitus (%)	16/38 (42.1)	13/31 (41.9)	3/7 (42.9)	1.04	0.179–5.51	0.964
Previous TIA/Stroke (%)	11/38 (28.9)	10/31 (32.2)	1/7 (14.3)	0.350	0.0170–2.45	0.36
On anticoagulation (%)	16/38 (42.1)	13/31 (41.9)	3/7 (42.9)	1.04	0.179–5.51	0.964
Smoker (%)	14/38 (36.8)	11/31 (35.5)	3/7 (42.9)	1.36	0.233–7.32	0.716
**Clinical presentation**						
NIHSS (IQR)	5 (4.00–7.75)	5 (4–7)	7 (3.5–11)	1.05	0.846–1.25	0.637
Lacunar localization (%)	16/38 (42.1)	12/31 (38.7)	4/7 (57.1)	2.11	0.399–12.4	0.378
IV-fluids given (%)	24/38 (63.2)	20/31 (64.5)	4/7 (57.1)	0.733	0.137–4.29	0.716
IV-antihypertensives given (%)	6/38 (15.8)	5/31 (16.1)	1/7 (14.2)	0.867	0.0410–6.86	0.904
**Hemodynamic characteristics**						
Heart rate, BPM (IQR)	77.5 (68–87.25)	78 (69.8–88.5)	62 (46.5–70.8)	0.970	0.918–1.02	0.219
Mean arterial blood pressure, mmHg (IQR)	105 (91.5–122.3)	108 (96.2–123)	89.1 (73.0–104)	0.944	0.887–0.989	0.0328*
Cardiac output, mL/min (IQR)	5.27 (4.29–6.67)	5.24 (4.19–6.62)	5.3 (4.7–6.73)	1.15	0.721–1.83	0.542
Cardiac stroke volume index, mL/m^2^ (IQR)	39.6 (31.5–48.4)	37.4 (30.9–47.7)	44.7 (42.3–55.3)	1.10	1.01–1.22	0.0468*
MCA mean flow velocity, cm/s (IQR)	38.4 (30.6–46.7)	39.9 (32.1–45.7)	34.4 (27.1–49.2)	0.983	0.909–1.05	0.617
Cardiac index L/min/m^2^ (IQR)	2.83 (2.36–3.26)	2.79 (2.34–3.25)	2.92 (2.88–3.27)	1.85	0.706–5.38	0.211
**(B) Multivariate**						
African American				0.093	0.002–1.15	0.112
Stroke Volume Index				1.14	1.03–1.31	0.0228*
Mean arterial pressure				0.925	0.851–0.981	0.0248*

Table shows patient demographic, clinical, and hemodynamic data for each variable. Continuous variables expressed as median (Interquartile range, IQR). Categorical variables expressed as count (percent,%). Table separated by **(A)** univariate and **(B)** multivariate logistic regression results expressed as odds ratio (OR) and 95% CI with P-values. *P < 0.05. ^&^P < 0.05 assessed by group comparisons.

### Clinical Presentation and Management

Baseline NIHSS was 5 (4–8) and was numerically higher in improved (7[5–11]) vs. unimproved patients (5[4–7]), but this difference was not statistically significant (*P* = 0.746). Four (57.1%) patients with neurological improvement vs. 12 (38.7%) patients without neurological improvement had lacunar strokes (*p* = 0.425). The rate of administration of IV-fluids and IV-antihypertensives in the ED were similar between patients that had or did not have neurological improvement.

### Hemodynamic Characteristics

**FIGURE 1:**
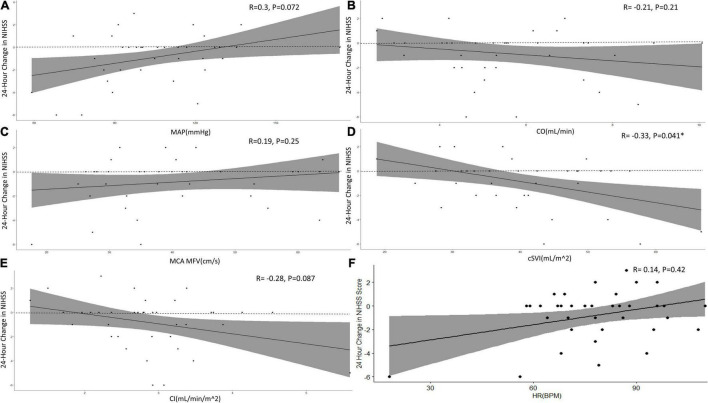
Correlations between hemodynamic measurements with 24-h change in NIHSS score. Figure shows Spearman-Rank correlations between hemodynamic parameters, **(A)** MAP, **(B)** CO, **(C)** MFV, **(D)** cSVI, and **(E)** CI with total 24-h NIHSS score change from baseline (dashed line). Line graphs are shown with error shaded in and respective *R*- and *P*-values. *P < 0.05.

MAP was significantly greater in those who did not improve compared to those that did improve. cSVI trended higher in those that improved vs. those that did not. CO, CI, MFV, and HR were similar between the no improvement vs. improvement group. Only cSVI statistically correlated with 24-h NIHSS change from baseline (Figure 1) demonstrating that a higher cSVI correlated with a greater reduction in NIHSS from baseline (*R* = -0.33, *P* = 0.041).

### Logistic Regression Analysis

**FIGURE 2:**
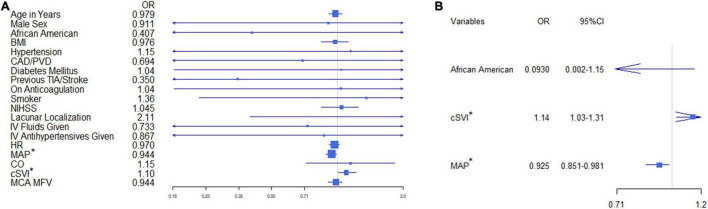
Univariate and multivariate odds ratio (OR) forest plots. Shows the OR forest plot for each variable with confidence intervals for **(A)** univariate and **(B)** multivariate models. *P < 0.05.

Only MAP and cSVI were statistically significant predictors for 24-h neurological improvement on univariate analysis (OR 0.944; 95% CI 0.877–0.989, *P* = 0.0328, and OR 1.1; 95% CI 1.01–1.22, *P* = 0.0468, Table 1A and Figure 2A). Step-wise logistic regression retained African American race, MAP and cSVI as variables for multivariate regression. Lower MAP and higher cSVI retained a statistically significant association with 24-h neurological improvement (OR 0.925; 95% CI 0.851–0.981, *P* = 0.0248 and OR 1.14; 95% CI 1.03–1.31, *P* = 0.0228). However, African American race did not reach statistical significance (OR 0.093; 95% CI 0.002–1.15, *P* = 0.112, Table 1B and Figure 2B). Pseudo-R^2^ for African American race, MAP and cSVI were 0.0195, 0.168, 0.133, while cumulative pseudo-R^2^ was 0.396.

## Discussion

### Independent Predictors Associated With Neurologic Improvement

In this prospective pilot study, we showed preliminary evidence for cSVI as an independent predictor for 24-h neurological improvement following AIS, correlating well with a larger reduction in NIHSS from baseline. Our findings add to the growing evidence associating cardiac function with AIS outcomes (Battaglini et al., 2020). AIS is associated with cerebrovascular autoregulation irregularities leading to significant blood pressure fluctuations, abnormal cerebral perfusion pressures, suboptimal penumbral perfusion and thus poorer neurological recovery (Reinhard et al., 2012). We found that MAP was an independent predictor of worsening 24-h neurological function; however, MAP along with CO and CI did not show a linear correlation with 24-h NIHSS change from baseline. Our findings corroborate with other studies, which have shown a lack of linear correlation between MAP and cerebral perfusion or post-AIS outcomes (Leonardi-Bee et al., 2002; Fuhrer et al., 2017; Rasmussen et al., 2020). Even though relatively lower BP (140–179 mmHg systolic) is associated with better neurologic outcomes post-stroke, the relationship between BP and has U shaped (Robinson et al., 1997; Leonardi-Bee et al., 2002). Extremely low BP (<120 mmHg systolic) post-stroke is rare, but at these ranges are in fact associated with worse outcomes (Leonardi-Bee et al., 2002). This is most likely caused by the failure of cerebral blood vessels to autoregulate efficiently to radical changes in BP. Cerebral perfusion pressure then becomes heavily dependent on parameters more associated with cardiac function (Tranmer et al., 1992).

### Autonomic Instability in Acute Ischemic Stroke and Cardiac Function

Autonomic imbalances, specifically reduced heart rate variability (HRV) and poor baroreceptor sensitivity, result in worse neurological function after AIS (Korpelainen et al., 1996; Robinson et al., 2003; Colivicchi et al., 2004; Xiong et al., 2018). Overall autonomic dysfunction assessed by Ewing battery, independently predicted worse 3-month functional outcomes in AIS patients (OR 3.26; 95% CI 1.14–9.34, *P* = 0.027) (Xiong et al., 2018). AIS, especially of the insular cortex or other cerebral structures that control heart function, results in both systemic and local sympathetic catecholamine release and also inflammation which can cause cardiac injury and dysfunction (Sposato et al., 2020). This in turn could result in worse cerebral perfusion to the penumbra leading to poorer neurological recovery. Therefore, it has been theorized that maintaining adequate cardiac function following AIS could improve neurological function (Fuhrer et al., 2017). Unfortunately, given the multifactorial effects on cardiac function in the setting of AIS, it is unknown as to which cardiac function parameter would be an adequate predictor for neurological recovery following AIS. To further complicate the issue, various hemodynamic variables do not respond to one another as they would under normal physiological conditions (Fuhrer et al., 2016).

Small preliminary studies did show a correlation between CO and cerebral blood flow in cerebral ischemic areas, but its role on neurological improvement has not been studied (Tranmer et al., 1992; Fuhrer et al., 2017). In this pilot study, we failed to find a strong association between CO or CI and neurological function, and there was a minimal difference in CO and CI in AIS patients that improved vs. those that did not improved. It is known that CO variability greatly increases following unopposed sympathetic activity during cholinergic blockade (Toska and Eriksen, 1993). Consequently, utilizing an absolute CO value may not be the most reliable indicator for cardiac function following autonomic dysregulation in AIS, but increased CO variability may indicate autonomic dysregulation and worse neurological prognosis. This coincides with our findings as CO and CI had significantly wide confidence intervals, possibly precluding their significance. CO variability in the setting of AIS warrants further study.

One would expect then that since CO = stroke volume x HR, decreased HRV would be compensated by an increase in stroke volume variability to maintain a consistent CO. Contrarily, though, in the setting of cholinergic blockade, cardiac stroke volume variability remains the same and does not equilibrate to the significant decrease in HRV (Akselrod et al., 1985; Toska and Eriksen, 1993). This most likely explains why CO, and therefore CI, variability increases following cholinergic blockade as all the CO variability will now be derived from the stroke volume variability (Elstad et al., 2011). Stroke volume variability most likely is independent to HRV because cardiac contractility is regulated by a different autonomic neural mechanism (Liu et al., 2004). In cats, it was shown that there are distinct cardiac ganglia found within the fat pad on the surface of the left ventricle (Gatti et al., 1997). This ganglion will selectively mediate any negative inotropic effect from vagal innervation to the left ventricle, independent from vagal stimulation to the sinoatrial node, which controls rate. Furthermore, there are significantly more post-ganglionic sympathetic nerves at the atria than the ventricles implicating less potential sympathetic damage to the ventricles than sinoatrial node following AIS autonomic dysregulation (Balint et al., 2019). Consequently, multiple studies have shown that autonomic changes to HRV did not correspond to changes in cardiac stroke volume variability (Toska and Eriksen, 1993; Akselrod et al., 2000; Liu et al., 2004). Thus, cardiac stroke volume variability is more likely influenced by mechanical factors based on Frank-Sterling’s Law, possibly making stroke volume a less variable cardiac parameter in the setting of autonomic dysregulation.

### Cardiac Stroke Volume Index Dynamics Following Acute Ischemic Stroke

Our study did show a possible association between cSVI and 24-h neurological function following AIS. Importantly, cSVI was the only parameter which had a linear association with 24-h NIHSS change from baseline. Cardiac stroke volume decreases significantly in patients with autonomic dysregulation and is a strong predictor for MFV in response to blood volume changes (Timmers et al., 2002; Fu et al., 2010; Bronzwaer et al., 2014). Furthermore, related literature shows that reduced left ventricular ejection fraction (LVEF), which is directly proportional to stroke volume, has been associated with worse longer-term functional outcome, though not with short-term outcomes, after adjusting for covariates (Mathias et al., 2013; Milionis et al., 2013; Byun et al., 2014). These findings indicate that inotropic status plays an important role in AIS functional outcomes. Post-AIS CT perfusion studies have shown that patients with reduced LVEF had larger hypoperfusion AIS lesion volumes (Garcia-Esperon et al., 2020). Although the exact pathophysiology behind this is unknown, one strong possibility is that patients with reduced LVEF have less blood flow to collateral circulation resulting in poorer perfusion to the penumbra (Hong et al., 2019; Garcia-Esperon et al., 2020). Since the extent of penumbra perfusion is a powerful predictor of post-AIS clinical outcomes, it is expected then that LVEF/stroke volume status would be a predictor for post-AIS neurological outcomes.

cSVI is equal to stroke volume divided by the body surface area, which allows for a more standardized comparison of stroke volume between patients regardless of body size. Therefore, cSVI maybe a more consistent and direct method to assess brain perfusion in the presence of autonomic dysregulation. More studies are required to investigate the mechanistic associations between cSVI and AIS.

## Limitations and Conclusion

A significant limitation to our preliminary study was the small sample size, in which the overall range of cardiac hemodynamic parameters and stroke severity was small. The study aimed to enroll patients as early as possible in their stroke care; however, given the challenges of confirming the diagnosis of stroke and requirements for informed consent, the final study cohort was smaller than anticipated and incorporated mild to moderate strokes. Furthermore, we did not assess long-term clinical outcomes, which carry greater weight than 24-h improvement. Nonetheless, 24-h improvement has been shown to strongly predict long term functional outcomes in AIS, thus 24-h improvement still holds significant prognostic importance (Takagi et al., 2014; Rangaraju et al., 2016). Additionally, we did not include patients who were treated with tPA, which is the mainstay treatment for AIS. However, more than 40% of patients present outside the treatment window for tPA and approximately 25% of tPA-eligible patients do not receive tPA due to late presentation or other contraindications. Therefore, there is still a significant clinical need to study AIS patients who do not receive tPA (Messé et al., 2016). Finally, our hemodynamic measurements only utilized non-invasive monitoring, without echocardiographic or invasive confirmation; still, the Nexfin device has shown in previous studies to provide reliable measurements of various hemodynamic parameters and is both quick to use and does not require extensive training before use (Broch et al., 2012; Martina et al., 2012). Given that the assessment and management of AIS must be quick and efficient, measurements of any pertinent clinical values must also be quick and efficient. Therefore, the Nexfin device has the potential to be readily utilized in the rapid assessment of AIS.

In this preliminary study assessing various hemodynamic parameters in AIS patients, we found cSVI and MAP to be associated with 24-h neurological improvement. Of which, cSVI was the only parameter which showed a linear association with NIHSS improvement from baseline. Therefore, cSVI is a unique parameter which warrants further study to determine its prognostic value and possible therapeutic implications.

## Data Availability Statement

The raw data supporting the conclusions of this article will be made available by the authors, without undue reservation.

## Ethics Statement

The studies involving human participants were reviewed and approved by the Henry Ford Hospital International Review Board. The patients/participants provided their written informed consent to participate in this study.

## Author Contributions

All authors affirm that the manuscript complies with author instructions, including author requirements. JM, CL, and PL applied for funding and designed the study. SC, RT, BN, and JM performed to data collection and analysis. JM and FC contributed equally to the creation of this manuscript. All other authors contributed to different aspects of manuscript preparation. This manuscript has not been published elsewhere. IRB approval was obtained for the study.

## Conflict of Interest

JM discloses that Edwards Lifesciences provided the Nexfin device at no cost but provided no other financial support. The Henry Ford Health System funded the study through an investigator initiated grant. PL discloses the receipt of unrelated research funding from Edwards Lifesciences. The remaining authors declare that the research was conducted in the absence of any commercial or financial relationships that could be construed as a potential conflict of interest.

## Publisher’s Note

All claims expressed in this article are solely those of the authors and do not necessarily represent those of their affiliated organizations, or those of the publisher, the editors and the reviewers. Any product that may be evaluated in this article, or claim that may be made by its manufacturer, is not guaranteed or endorsed by the publisher.

## Abbreviations

NIHSS: National Institute of Health Stroke Scale
AIS: acute ischemic stroke
ED: emergency department
MAP: mean arterial pressure
CO: cardiac output
CI: cardiac index
cSVI: cardiac stroke volume index
TCD: transcranial Doppler
MCA: middle cerebral artery
MFV: mean flow velocity
IV: intravenous
CAD/PVD: coronary artery or peripheral vascular disease
HRV: heart rate variability
TIA: transient ischemic attack.
